# Habitat-Specific Locomotor Variation among Chinese Hook Snout Carp (*Opsariichthys bidens*) along a River

**DOI:** 10.1371/journal.pone.0040791

**Published:** 2012-07-19

**Authors:** Shi-Jian Fu, Zuogang Peng, Zhen-Dong Cao, Jiang-Lan Peng, Xiao-Ke He, Dandan Xu, An-Jie Zhang

**Affiliations:** 1 Laboratory of Evolutionary Physiology and Behavior, Chongqing Key Laboratory of Animal Biology, Chongqing Normal University, Chongqing, China; 2 The Key Laboratory of Freshwater Fish Reproduction and Development (MOE), Southwest University School of Life Science, Beibei, Chongqing, China; University of Roehampton, United Kingdom

## Abstract

The Wujiang River is a tributary of the upper Yangtze River that shows great variations in its flow regime and habitat condition. Dams have been built along the Wujiang River and have altered the habitats profoundly enough that they may give rise to reproductive isolation. To test whether the swimming performance and morphology of the Chinese hook snout carp (*Opsariichthys bidens*), varied among habitats and whether the possible differences had a genetic basis, we measured the steady and unsteady swimming performance, external body shape and genetic distance among fish collected from both the main and tributary streams of the upper, middle and lower reaches along the river. We also measured the routine energy expenditure (RMR), maximum metabolic rate (MMR), cost of transport (COT) and calculated the optimal swimming speed. The steady swimming capacity, RMR, MMR and optimal swimming speed were all higher and the COT was lower in the upper reach or tributary streams compared with the lower reach or main stream. However, unsteady swimming performance showed no variation among collecting sites. Flow regimes as suggested by river slope and water velocity were positively correlated with steady swimming performance but not with unsteady swimming performance. Predation stress were significantly related with body morphology and hence energy cost during swimming but not *U*_crit_ value. The fish from only one population (Hao-Kou) showed relatively high genetic differentiation compared with the other populations. Fish from the upper reach or tributary streams exhibited improved steady swimming performance through improved respiratory capacity and lower energy expenditure during swimming at the cost of higher maintenance metabolism. There was no correlation between the steady and unsteady swimming performance at either the population or the individual levels. These results suggest that a trade-off between steady and unsteady swimming does not occur in *O. bidens*.

## Introduction

Fish inhabit environments that vary greatly in the intensity of water velocity and predation stress, and these habitat conditions are generally believed to be of major evolutionary significance [1]. For most fish, swimming behaviors are essential for carrying out countless tasks. Thus, natural selection is predicted to favor different swimming capabilities under different habitats and consequently to drive major patterns of phenotypic variation in fishes [1]. Swimming performance can be classified as steady or unsteady swimming performance. In nature, steady swimming is commonly employed during various activities, such as holding station in a water current, seeking favorable abiotic conditions or migration [2], [3] whereas unsteady swimming is common during social interactions, predator avoidance and navigating in structurally complex environments [4], [5]. In fish, the critical swimming speed (the water speed at which a fish can no longer maintain its position or its maximum sustainable swimming speed, *U*_crit_) is widely used to evaluate steady swimming performance [2], [6], [7] whereas fast-start performance (brief, sudden accelerations used by fish during predator–prey encounters) is usually used to evaluate unsteady swimming performance [8]–[10]. It is generally believed that the body morphology necessary to maximize steady swimming efficiency involves great depth in anterior body and a shallow caudal region whereas maximal unsteady swimming efficiency involves the opposite characteristics [1]. Thus, a body morphology optimized for burst speed will be obtained at the cost of reduced performance at steady swimming [11]. This general trade-off between steady and unsteady swimming performance has long been hypothesized to play an important role in the ecology and evolution of fish. Scientists suggested that both genetic divergence and phenotypic plasticity stemmed from this trade-off may lead to phenotypic differentiation and generate macroevolutionary patterns across flow regimes and predation condition [11]–[14].

Selection pressures on locomotor performance in different habitats drive differences in morphology and locomotion in different flow regimes. The trade-off is expected to favor steady swimming in high-flow environments, where fish must often swim to maintain position and perform routine tasks, but unsteady swimming in low-velocity environments, where fish are largely freed from severe demands on endurance and can instead exploit strategies requiring high acceleration or maneuverability [1]. Trade-off between steady and unsteady swimming underlies predator-driven divergence also demonstrated in fish such as *Gambusia affinis*. Such divergent natural selection between alternative flow regime and predation environments often generates and maintains morphological and physiological phenotypic diversity [15]–[17]. However, despite the volume of literature, we still lack an understanding of the major patterns of the effects of water velocity and predation stress on fish phenotypes. Furthermore, too few studies have addressed these topics or conducted analyses within a phylogenetic framework.

*Opsariichthys bidens* is one of the most widely distributed small Asiatic cyprinids. It generally occupies fast-flowing mountainous steams. It lives for no more than 2 years in the field, and its short life history may favor microevolution and gene assimilation. The Wujiang River is the largest tributary stream of the upper Yangtze River, with a length of 1037 km. The average river slope varies from 12.9‰ in the upper reach to 0.62‰ in the lower reach. Thus, the flow regime and predation stress changes profoundly along the river (Table 1). Since 1970, numerous dams have been built along the main and tributary streams. These changes may produce reproductive isolation and hence accelerate the microevolution of this species. Thus, the aim of this study is to test whether the locomotor performance and morphology of *O. bidens* vary along the river and whether the possible changes are correlated with genetic distance. To test our hypothesis, we collected one-year-old female *O. bidens* from both the main stream and the tributary streams in the upper, middle and lower reaches. We chose females because they are easier to capture in the field and exhibit less variation in body size than the males. We examined the differences in body shape, genetic distance, and critical swimming speed (*U*_crit_) as indicators of steady swimming performance and fast-start escape performance as an indicator of unsteady swimming performance among populations of *O. bidens* along the Wujiang River.

**Table 1:** Overview of environmental parameters at the five collection sites.

	Lower reach		Middle reach		Upper reach
	Main stream	Tributary stream	Main stream	Tributary stream	Main stream
Collecting sites	Yang-Jiao (I, YJ)	Hao-Kou (IV, HK)	Si-Nan (II, SN)	San-Du (V, SD)	Da-Guan (III, DG)
Coordinate	N29 °23′48′′,E107 °36′49′′	N29 °02′32′′,E107 °50′43′′	N27 °56′44′′,E108 °15′44′′	N27 °39′35′′,E107 °18′51′′	N26 °51′44′′,E106 °10′03′′
Altitude (m)	173	320	362	780	859
River width (m)	100∼200	30∼50	100∼200	20∼30	30∼50
Distance from river mouth (km)	50	115	350	650	700
Water temperature (°C)	26.0	25.0	23.8	21.5	21.3
River slope (‰)	0.62	4.08	0.97	5.30	3.65
Water velocity (m s^−1^)	1.10	1.90	1.45	1.82	2.32
Predators species	*Silurus meridionalis, Siniperca kneri*	*Silurus meridionalis, Siniperca kneri*	*Silurus meridionalis, Erythroculter ilishaeformis*	*Silurus asotus*	*Silurus asotus, Channa argus*
Size of predators (g)	1,243	1,020	826	325	435
Abundance of predators (%)	15.1	6.1	3.3	2.6	4.5
Dissolved oxygen level(% saturate)	102.0	103.0	82.5	98.7	97.9

## Materials and Methods

### Ethics Statement

The fish collection and field investigation were under the permission of the fisheries administrative institution of Chongqing City, Guizhou Province and Yunnan Province. They are responsible for the regulation of local fishes in the Wujiang River. Our project was also approved by the National Natural Science Foundation of China (No. 31172096) and Chongqing Normal University.

### Collecting Sites and Fish Collection

**Figure 1:**
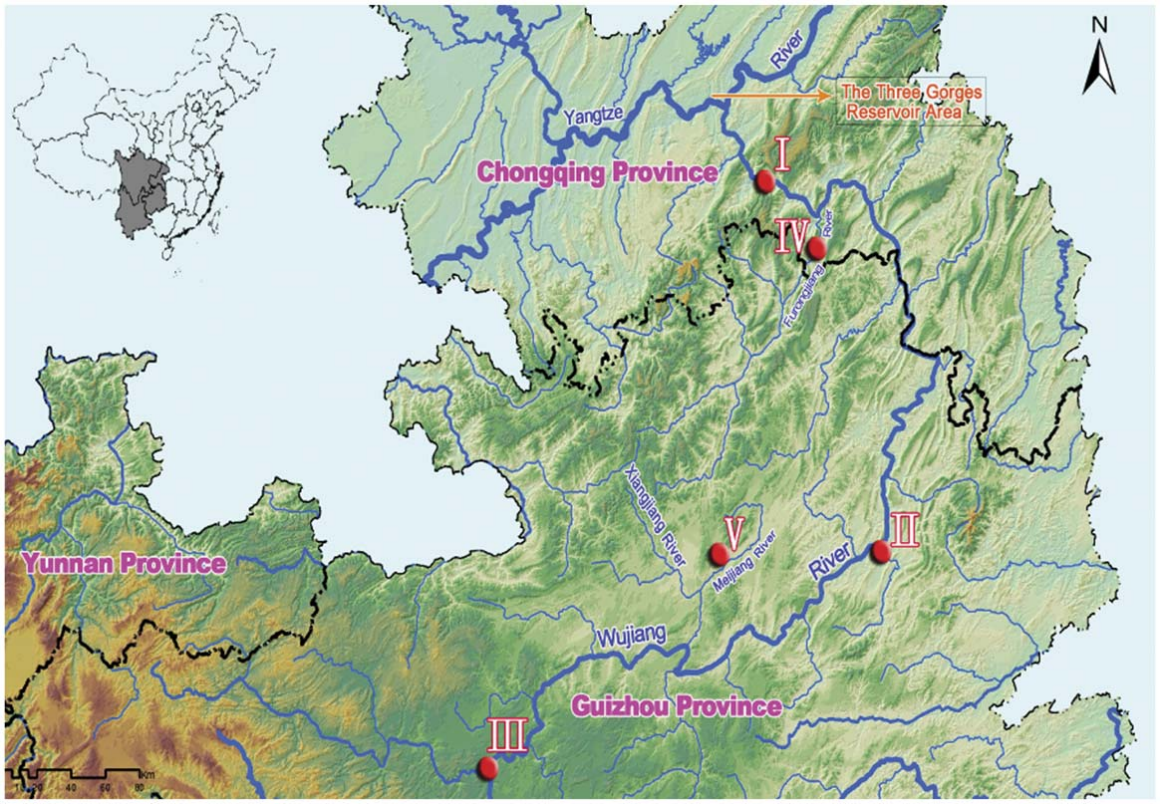
The location map of collecting sites (I: Yang-Jiao, II: Si-Nan, III: Da-Guan, IV: Hao-Kou, V: San-Du, see **Table 1** for more information).

We planned to collect Chinese hook snout carp from three pairs of sites (i.e., six populations) along the Wujiang River, i.e., the upper, middle and lower reaches (Fig. 1). One site was within the main stream for each pair, and the other site was in a tributary stream. However, we did not collect any individuals from the tributary stream of the upper reach. The information on each collecting site is shown in Table 1. We collected *O. bidens* by hook-and-line angling and seine from each site. We only used one year adult female fish whose body length (BL) was less than 10 cm (8.12±0.27 cm, n = 36). We use one year adult female because it is easier to be caught in field. We use fish less than 10 cm because we want the body size of experimental fish to be similar (the usual size of adult female *O. biden* is 40–100 mm).

We loaded and transported all the experimental equipment in two trucks during the entire field experiment. After we collected the fish, we reared them in a fully aerated and thermoregulated tank (100 L) with the same temperature (±1°C) as that in the field of each collecting sites. Water was transferred from the river and filtered through filter cotton. After two days of fasting, each fish was first transferred to the fast-start equipment [18] for the measurement of unsteady swimming. The fish was then transferred to a Brett swimming channel for *U*_crit_ and oxygen consumption rate (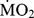) measurement after a recovery period of at least 8 h. The fish was then euthanized with an overdose of tricaine methanesulfonate (MS-222) for the measurement of body shape. After the measurement of body shape, the fish was stored in a 70% ethanol solution for approximately 1 week and was then transferred to a 95% ethanol solution for later phylogenetic analysis. We used the same fish for different measurements because we sought to determine the relationship between different traits at the individual level and because a pilot experiment found that the fast-start measurement had no effect on the subsequent *U*_crit_ measurement.

### Measurement of Fast-start Performance

Each fish was acclimated for 48 h without food in the experimental tanks at the same temperature as in the field. A small white plastic ball (diameter: 1 mm) was then attached to the dorsal side of each fish at the center-of-mass (CM) position determined by a pilot experiment after the fish was slightly anesthetized with MS-222 (50 mg L^−1^) [19]. Single fish were then transferred to the experimental glass tank. A reference grid of 1 cm squares was attached to the floor of the tank. Black paper covered the sides of the experimental tank so that the fish could not see the approaching stimulus. The fish was introduced into the acclimation zone of the fast-start experimental system and allowed to rest for 1 h. The depth of water in the tank was 10 cm. After 1 h acclimation, the fish was introduced from acclimation zone to the filming zone through the passage way (fish moved to the filming zone spontaneously at most cases, if not they were driven into the filming zone gently by hand net). The escape responses were elicited with a manually triggered electrical impulse (0.5 V cm^−1^; 10 ms, as determined by a previous study [19] when the fish was holding position at the center of the filming zone. A high-speed camera (Basler A504K; 500 frames s^−1^) was used to record the entire time course of the fast-start experiment (time span: 3 s). The initiation of the experiment was recorded as soon as the LED became illuminated (0 ms). The coordinates of the CM were measured with an E-Ruler. The following parameters were calculated: response latency (*t*, ms), maximum linear velocity (*V*_max_, m s^−1^) and turning radius (*r*, mm). The response latency *t* was defined as the time between the initiation of the stimulus (LED illumination) and the time when escape behavior was observed.

**Table 2:** The fast-start performance of fish collected from different sites (Means±SD).

	n	Maximum velocity (m s^−1^)	Response latency (ms)	Turning radius (mm)
Yang-Jiao	6	1.42±0.24	12.3±5.1	9.9±4.7
Hao-Kou	6	1.24±0.42	12.7±5.9	10.9±3.9
Si-Nan	8	1.35±0.28	12.8±11.9	15.3±10.2
San-Du	8	1.28±0.25	12.0±9.9	14.0±8.8
Da-Guan	7	1.25±0.24	10.7±2.9	11.2±3.2
Statistical results (*P*-values)				
Reach effect		0.632	0.787	0.465
Stream effect		1.397	0.574	0.539
Interaction effect		0.147	0.386	0.298

**Table 3:** The Pearson’s correlation between ecological parameters and locomotor and morphological parameters.

	River slope	Water velocity	Temperature	Altitude	Size of predators	Abundance of predators	Dissolved oxygen level
Response latency	r = −0.214, *P* = 0.257	r = −0.143, *P* = 0.450	r = 0.208, *P* = 0.270	r = −0.195, *P* = 0.302	r = 0.219, *P* = 0.245	r = 0.116, *P* = 0.543	r = −0.086, *P* = 0.653
Maximum linear velocity	r = −0.125, *P* = 0.512	r = −0.150, *P* = 0.430	r = 0.054, *P* = 0.777	r = −0.084, *P* = 0.658	r = 0.050, *P* = 0.792	r = 0.058, *P* = 0.764	r = −0.092, *P* = 0.629
Turning radius	r = −0.116, *P* = 0.543	r = 0.058, *P* = 0.762	r = −0.091, *P* = 0.632	r = 0.088, *P* = 0.645	r = −0.067, *P* = 0.727	r = −0.139, *P* = 0.463	r = −0.266, *P* = 0.155
Critical swimming speed	r = 0.518, *P* = 0.003*	r = 0.408, *P* = 0.025*	r = −0.069, *P* = 0.715	r = 0.146, *P* = 0.441	r = −0.096, *P* = 0.614	r = 0.030, *P* = 0.875	r = 0.618, *P*<0.001*
Maximum metabolic rate	r = 0.368, *P* = 0.045*	r = 0.570, *P* = 0.001*	r = −0.322, *P* = 0.083	r = 0.412, *P* = 0.024*	r = −0.293, *P* = 0.117	r = −0.329, *P* = 0.076	r = 0.102, *P* = 0.591
Fitness ratio	r = −0.331, *P* = 0.074	r = −0.471, *P* = 0.009*	r = 0.757, *P*<0.001*	r = −0.663, *P*<0.001*	r = 0.752, *P*<0.001*	r = 0.746, *P*<0.001*	r = 0.497, *P* = 0.005*
Aspect ratio	r = 0.011, *P* = 0.956	r = −0.096, *P* = 0.614	r = 0.175, *P* = 0.356	r = −0.178, *P* = 0.348	r = 0.154, *P* = 0.415	r = 0.095, *P* = 0.617	r = 0.119, *P* = 0.530
Caudal peduncle depth factor	r = −0.255, *P* = 0.174	r = −0.325, *P* = 0.079	r = 0.610, *P*<0.001*	r = −0.506, *P* = 0.004*	r = −0.724, *P*<0.001*	r = −0.725, *P*<0.001*	r = −0.392, *P* = 0.032*

* significantly different (*P*<0.05).

### Measurement of *U*_crit_ and 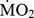


After the fast-start measurement, the fish was transferred to the swimming respirometer [20]. A Brett-type swimming tunnel respirometer was used to measure the fish’s *U*_crit_. For details see previous papers [20]–[22]. The fish were individually transferred into the swim tunnel and allowed to recover for 8 h. During this recovery period, aerated water flowed continuously through the respirometer. The water temperature in the swimming chamber was controlled by a water bath connected to a stainless steel heat exchanger. The water velocity increased in 6 cm s^−1^ increments every 30 minutes until the fish became fatigued. Fatigue was defined as the time at which the fish failed to move off the rear honeycomb screen of the swimming chamber for 20 s [7]. *U*_crit_ was calculated for individual fish using Brett’s equation [6]:
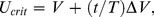
(1)where *V* is the highest speed at which the fish swam for the full time period (cm s^−1^), *ΔV* is the velocity increment (6 cm s^−1^), *T* is the prescribed period of swimming per speed (30 min) and *t* is the time that the fish swam at the final speed (min). The swim tunnel was designed to switch between a closed mode and an open mode. The closed mode was used for respirometry, and the open mode was used to replenish the oxygen. A small volume of water was drawn from the sealed respirometer by a peristaltic pump, forced past a dissolved oxygen probe housed in a sealed temperature-controlled chamber, and then returned to the respirometer. The oxygen concentration (mg L^−1^) was recorded once every 2 min. The 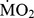 (mg kg^−1^ h^−1^) of an individual fish during swimming was calculated from the depletion of oxygen according to the equation
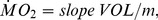
(2)where slope (mg L−1 h−1) is the decrease in the water’s dissolved oxygen content per minute, VOL is the total volume of the respirometer (3.5 L) minus the volume of the fish and m is the body mass (kg) of the fish. The slope was obtained from a linear regression between time (min) and the water’s dissolved oxygen content (mg L−1); only slopes with an r2>0.95 were considered in the analysis. The 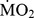 was adjusted to a standard body mass of 1 kg with a body mass coefficient of 0.75. The routine metabolic rate (RMR) was calculated using the fitting equation between 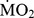 and swimming speed to obtain the 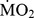 at 0 cm s−1. The maximal observed 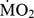 during the Ucrit test was defined as the maximum 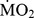 (MMR).

**Figure 2:**
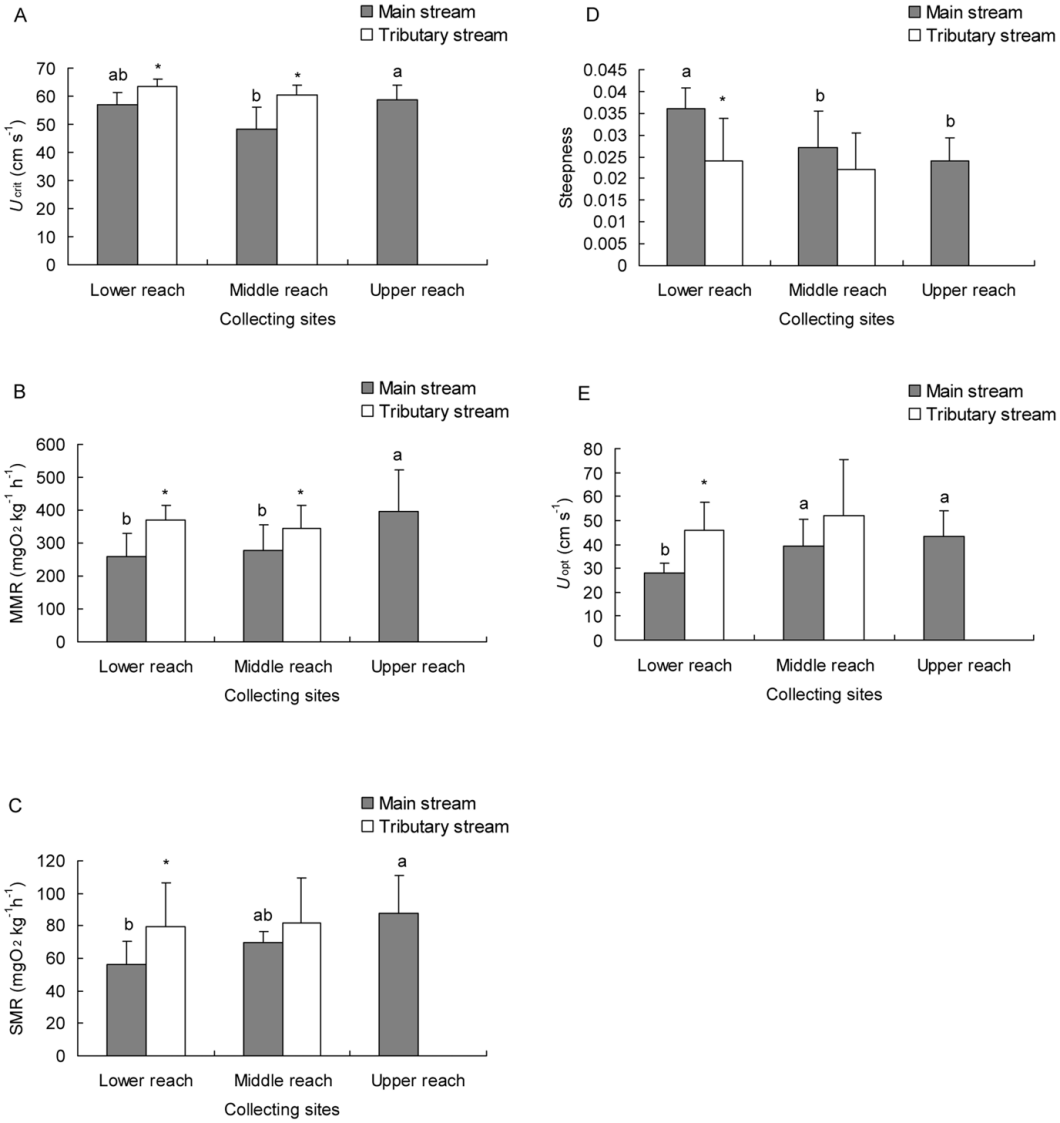
The critical swimming speed (*U*_crit_) (A), maximum metabolic rate (MMR) (B), routine metabolic rate (RMR) (C), steepness (D) and optimal swimming speed (*U*_opt_) (E) of fish collected from different sites along the Wujiang River. a, b, c, Values not sharing a common letter suggests significant difference (*P*<0.05);* indicates significant difference between main and tributary streams.

**Figure 3:**
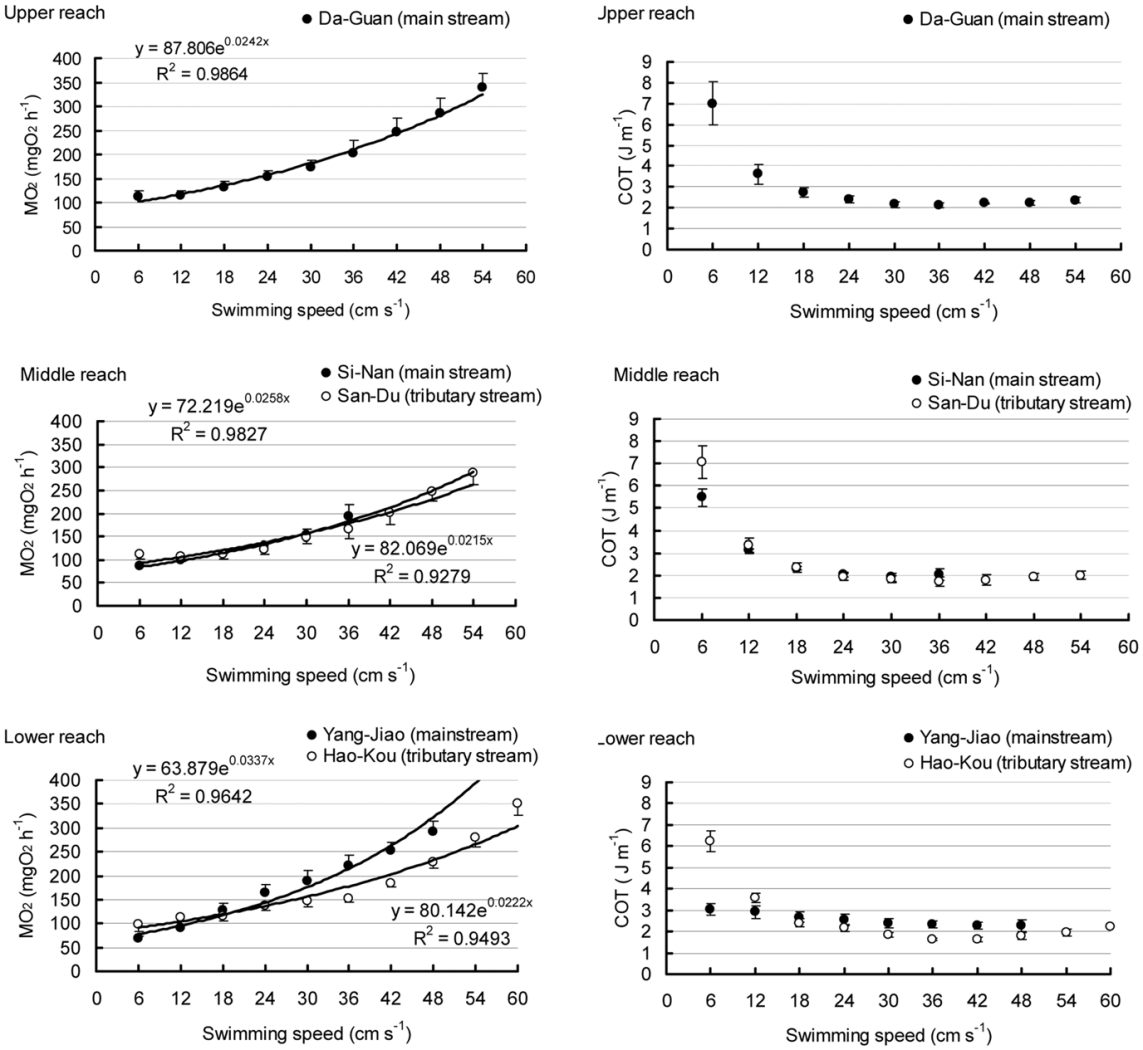
The metabolic rate (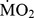) and cost of transport (COT) under different swimming speed of fish collected from different collecting sites.

**Table 4:** The effect of swimming speed, different reaches and streams on metabolic rate (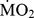) and cost of transport (COT) of fish collected from different sites.

		Metabolic rate		Cost of transport	
	df	F	Sig.	F	Sig.
Speed effect	10	84.947	0.000*	70.083	0.000*
Reach effect	2	1.136	0.323	4.128	0.017*
Stream effect	1	12.687	0.000*	0.028	0.867
Speed×Reach	19	0.462	0.975	3.736	0.000*
Speed×Stream	9	2.648	0.006*	8.149	0.000*
Reach×Stream	1	1.811	0.180	0.021	0.885
Speed×Reach×Stream	8	0.542	0.824	1.236	0.278

* significantly different (*P*<0.05).

**Table 5:** Comparison of locomotion parameters between tributary and main streams and among upper, middle and lower reaches.

	Reach effect	Stream effect	Interaction effect
Critical swimming speed	*P* = 0.019*	*P* = 0.001*	*P* = 0.246
Maximum metabolic rate	*P* = 0.013*	*P* = 0.049*	*P* = 0.118
Routine metabolic rate	*P* = 0.050*	*P* = 0.036*	*P* = 0.481
Steepness	*P* = 0.032*	*P* = 0.048*	*P* = 0.186
Optimal swimming speed	*P* = 0.142	*P* = 0.010*	*P* = 0.645

The routine metabolic rate, steepness and optional swimming speed are calculated from the fitted parameters in equation: Y = α e^βX^.

* significantly different (*P*<0.05).

**Table 6:** The morphology of fish collected from different sites (Means±SD).

	Yang-Jiao	Hao-Kou	Si-Nan	San-Du	Da-Guan
n	6	6	8	8	7
Fitness ratio (BL/BD)	5.31±0.39	5.05±0.17	4.40±0.31	4.37±0.14	4.39±0.29
Aspect ratio (CFH^2^/Area)	4.12±0.61	4.22±0.69	3.99±0.62	4.05±0.34	3.96±0.34
Caudal peduncle depth factor (CPD/BD)	43.9±3.2	42.7±1.5	38.5±3.1	38.3±2.3	39.4±2.6

BL: body length, DB: body depth, CFH: caudal fin height, CPD: caudal peduncle depth.

### Shape Measurement Methods

Geometric morphometric methods were used to quantify body shape. Photographs of the right side of each specimen (n = 39) viewed together with a ruler were taken with a digital camera and then analyzed with the thin plate spline (tps) software package (http://life.bio.sunysb.edu/morph). These programs provide various types of statistical analyses using partial warp scores as shape variables and/or expressing the results of a morphometric analysis as a shape deformation. We used tpsDig2 (a program for digitizing landmarks and outlines for geometric morphometric analyses) and tpsUtil for data acquisition and editing. After first concatenating all photographs into a single file, we placed landmarks on 17 morphological features in each image (Fig. S1). We then used tpsSuper to perform a least-squares orthogonal generalized Procrustes analysis and plot a consensus configuration. We then used tpsReg performs a multivariate multiple regression of shape (as captured by partial warp scores and the uniform shape component) onto the independent variable, i.e., the collecting site, ecological parameters, *U*_crit_ value and *V*_max_ value.

We also measured the body length, body height, caudal fin area, caudal fin height, and caudal peduncle height and calculated the values of the following morphological traits:

Relative caudal peduncle height (CPH, %) = 100×caudal peduncle height/body length,

Caudal fin aspect ratio (AR) = caudal fin height^2^/caudal fin area,

Fitness ratio (FR) = body length/body height.

### Genetics

#### DNA extraction, PCR amplification and sequencing

Total genomic DNA was extracted from muscle tissue using the QIAGENE DNeasy Tissue Kit (Qiagen, Germany) following the manufacturer’s protocol. The mitochondrial cyt b gene was amplified with the primer set L14724 (5′-GACTTGAAAAACCACCGTTG-3′) and H15915(5′-CTCCGATCTCCGGATTACAAGAC-3′) [23] by the polymerase chain reaction (PCR) in 50 µl reactions containing 4 µl of dNTPs (2.5 mM), 5 µl of 10×reaction buffer (Mg^2+^ free), 3 µl of MgCl_2_ (25 mM), 1 µl of each primer (10 µM), 0.2 µl (2.5 U) of Taq DNA Polymerase (rTaq, Takara), and 2 µl of template DNA. Reaction conditions were 2 min at 94°C, followed by 30 cycles of 30 s at 94°C, 30 s at 56°C, 90 s at 72°C, and a final 6 min at 72°C. PCR products were purified and sequenced by a commercial company using the same primer set, L14724 and H15915.

#### Sequence alignment and phylogenetic analysis

Nucleotide sequences were aligned using the Clustal X multiple alignments program [24] with the default parameters. The Kimura 2-parameter genetic distances were analyzed using MEGA5 [25].

### Statistics

All values are presented as the means ± S.D., and *P*<0.05 was used as the level of statistical significance. The multivariate multiple regression of shape onto any parameters was performed with tpsReg (http://life.bio.sunysb.edu/morph). STATISTICA 4.5 was used for other data analysis except tps. The relationship between ecological parameters (water velocity, river slope, water temperature and altitude) and locomotor (*U*_crit_, *V*_max_, *r* and *t*) and morphological parameters (CPH, FR and AR) were analyzed with Pearson’s correlation. The differences in measured morphological traits, *U*_crit_, MMR and all variables involved in fast-start performance among different collecting sites (i.e., between streams and among reaches) were analyzed with a two-way analysis of variance (ANOVA). The effect of swimming speed and collecting sites on 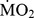 and the cost of transport (COT) was analyzed with a three-way (between streams, among reaches and among speeds) ANOVA. The ANOVA was followed by a Duncan multiple comparison test if it was necessary to evaluate the differences between the values of the different experimental groups. Nonlinear estimation (exponential equation) was also used to describe the relationship between 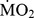 and swimming speed.

## Results

### Swimming Performance

#### Fast-start performance

Body length had no effect on any variables involved in fast-start performance in this study (Pearson correlation, *P* = 0.443–0.895). None of the variables involved in fast-start performance differed significantly among the fish collected from different collecting sites (Table 2). Furthermore, none of the variables involved in fast-start performance showed significant correlation with any ecological parameters (Table 3).

***U*** **_crit_**. Body length had no effect on the absolute *U*_crit_ expressed as cm s^−1^ (Pearson correlation coefficient r = −0.188, *P* = 0.287) but was negatively related to the body-length-adjusted *U*_crit_ (i.e., the relative *U*_crit_) expressed as BL s^−1^ (Pearson correlation coefficient r = −0.913, *P*<0.001). Thus, we used the absolute *U*_crit_ in this study. A two-way ANOVA showed that the *U*_crit_ (i.e., the absolute *U*_crit_ here and throughout the manuscript) changed significantly among different reaches (*P* = 0.002) and between the tributary and the main stream (*P*<0.001) (Fig. 2A). The *U*_crit_ of the fish collected from the middle reaches of the main stream (Si-Nan, SN) was significantly lower than the value from the upper reaches (Da-Guan, DG) (*P* = 0.001). The *U*_crit_ of the fish collected from tributary streams was significantly higher than that of those collected from the main stream within each reach, i.e., HK > YJ (*P* = 0.048) and San-Du (SD) > SN (*P*<0.001). The *U*_crit_ was positively related with water velocity (*P* = 0.025), river slope (*P* = 0.003) and water dissolved oxygen level (*P*<0.001) (Table 3).

#### Trade-off between steady and unsteady swimming performance

There was no significant correlation between *U*_crit_ and *V*_max_ at either the individual (n = 34, r = 0.047, *P* = 0.808) or the population (n = 5, r = 0.367, *P* = 0.544) levels. There was also no significant correlation between *U*_crit_ and any other fast-start variables (Pearson correlation, *P* = 0.444∼0.996).

#### MMR

The MMR exhibited significant changes among different reaches (*P* = 0.007) and between the tributary and main streams (*P* = 0.008) (Fig. 2B). In the main stream, the MMR of the fish collected from the upper reaches was significantly higher than those collected from both the middle (*P* = 0.009) and lower reaches (*P* = 0.005), but there was no significant difference in the MMR values between the fish collected from the middle and lower reaches (*P* = 0.667). The MMR of the fish collected from the main stream was significantly lower than that from the tributary stream in both the lower reach (*P* = 0.013) and the middle reach (*P* = 0.048). The MMR was positively related with both water velocity (*P* = 0.001), river slope (*P* = 0.045) and altitude (*P* = 0.024).

#### The swimming 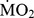 and cost of transport (COT)

The 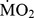 increased significantly with swimming speed in all fish groups (*P*<0.001) and changed significantly between the tributary and main streams (interaction: *P* = 0.006) but not among the different reaches (Fig. 3, Table 4). The 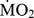 values of the tributary stream were higher than those of the main stream at a low swimming speed but lower than the main stream values at a high swimming speed. The relationship between the 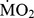 (Y) and swimming speed (X) could be described with the equation.
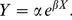
(3)


The parameter α could be interpreted as the RMR whereas the parameter β could be interpreted as the steepness of the power curve and, hence, the energy cost of swimming. The optimal swimming speed (*U*_opt_), at which the fish showed the minimum swimming energy cost, was given by 1/β. The RMR values for the fish collected from the lower reach and the main stream were significantly lower than the RMR values from the upper reach or the tributary streams whereas the steepness values for the fish collected from the lower reach and the main stream were significantly higher than the steepness values from the upper reaches or the tributary streams (*P*<0.05) (Fig. 2C–D, Table 5). The optimal *U*_crit_ exhibited a significant difference between the tributary and the main stream (*P* = 0.010) (Fig. 2E).

Unlike the 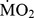, the COT decreased significantly with swimming speed in all fish groups (*P*<0.001) and changed significantly among different reaches (interaction effect: *P* = 0.017) but not between the tributary and main streams (*P* = 0.867) (Fig. 3). The upper reach showed a significantly higher COT at a lower swimming speed. The main stream showed a significantly lower COT at a low swimming speed but a higher COT at a high swimming speed.

### Morphology

The relative warp visualization plots for the fish collected from different sites were shown in Fig. S1. The landmarks showed no variation between streams and among reaches. However, tpsReg suggested that morphology was closely related with all ecological parameters except dissolved oxygen level (abundance of predator, F_34, 981_ = 3.29, *P*<0.001; size of predator, F_34, 981_ = 3.14, *P*<0.001; water temperature, F_34, 981_ = 2.98, *P*<0.001; altitude, F_34, 981_ = 2.81, *P*<0.001; water velocity, F_34, 981_ = 2.19, *P*<0.001; river slope, F_34, 981_ = 1.87, *P* = 0.002; dissolved oxygen level, F_34, 981_ = 1.10, *P* = 0.321). Furthermore, all measured morphological variables (i.e., FR, AR and CPDF) showed no significant variation among the different collecting sites if body length was used as a covariate (Table 6). A further ANOVA testing only the mainstream collection sites (YJ, SN and DG) found that FR of fish collected from YJ were significantly larger than those from SN and DG. FR was positively correlated with water velocity (*P* = 0.009), water temperature (*P*<0.001), altitude (*P*<0.001), size of predators (*P*<0.001), abundance of predators (*P*<0.001) and dissolved oxygen level (*P* = 0.005). Caudal peduncle depth factor was positively correlated with water temperature (*P*<0.001), altitude (*P* = 0.004), size of predators (*P*<0.001), abundance of predators (*P*<0.001) and dissolved oxygen level (*P* = 0.032) while aspect ratio showed no significant correlation with any of ecological parameters.

There was no significant correlation between any swimming variable (i.e., *U*_crit_, *V*_max_) and the morphological traits (i.e., FR, AR and CFAR). However, tpsReg suggested that morphology was significantly related with *U*_opt_ (F_34, 981_ = 2.98, *P*<0.001), steepness (F_34, 981_ = 2.83, *P*<0.001), RMR (F_34, 981_ = 2.41, *P*<0.001) and *V*_max_ (F_34, 981_ = 1.60, *P = *0.017), but not *U*_crit_ (F_34, 981_ = 0.63, *P* = 0.948) and *r* (F_34, 981_ = 0.89, *P* = 0.651).

### Genetics

The pairwise sequence divergence among the 39 *O. bidens* individuals varied from 0 to 2.37%, with a mean of 0.60%. We found that the *O. bidens* from the Hao-Kou (HK) population exhibited the highest genetic divergence compared with the other populations of the same species (within- group mean distance: 1.26% [HK population, 6 individuals] vs. 0.41% [the remaining *Opsariichthys bidens*]).

## Discussion

In this study, we aimed to investigate the intra-species variation of locomotor performance in *O. bidens* and its relationship with morphology and genetic distance. We clearly demonstrated a marked divergence in steady swimming performance but not in unsteady swimming performance among different populations. The improved steady swimming performance in the upper reach and the tributary streams was in part a result of increased respiratory capacity and lower swimming cost due to morphological difference. The difference in swimming performance was not accompanied by a genetic difference since only the fish from HK showed relatively high genetic differentiation compared with the other populations. A trade-off between steady and unsteady swimming performance was not found at either the individual or the population levels.

### Swimming Performance

The *U*_crit_ changed markedly along the Wujiang River, with a 24% change in *U*_crit_ between HK and SN. Fish from the tributary streams exhibited higher *U*_crit_ values than fish from the main stream, and fish from the upper reach (i.e., DG) of the main stream also exhibited higher *U*_crit_ values than fish from the middle reach (i.e., SN). These results agreed with our prediction, i.e., fish from the upper reach and the tributary streams usually have higher *U*_crit_ values than fish from the lower reach and the main stream because the selection will favour steady swimming in high water velocity and low-predation environments, but instead favour unsteady swimming in low water velocity and high-predation environments. In this study, *U*_crit_ was closely related to ecological parameters such as river slope and water velocity but not size and abundance of predators. It suggested that flow regime rather than predation stress drives the variation of steady swimming performance among different habitats. The habitat-specific *U*_crit_ variation had been documented in other fish species [26]–[28]. In these previous studies, the different fish groups analyzed were usually found in habitats that differ substantially in predation stress, flow regime and physical distance because they were associated with different water bodies. To our knowledge, this study is the first to find a substantial divergence in *U*_crit_ values among fish living within a single water body. Most published studies that demonstrated variation in *U*_crit_ also showed marked concurrent morphological changes. The morphological changes are usually assumed to have profound effects on the swimming transport cost and therefore on the maximum swimming speed [29]. The morphological change also showed in *O. bidens*, but the change was more closely related to predation stress than other physical environmental factors of habitats. The variation of body shape showed no effect on maximum steady swimming speed as suggested by *U*_crit_, but altered the swimming energy cost profoundly as suggested by the significant relationship between body shape and steepness, *U*_opt_ and maintenance metabolism (i.e. RMR) (see details in the next paragraph). In this study, the improved *U*_crit_ was not due to morphological variation. However, our examination of the variation in the MMR among different collecting sites showed a 35% variation in the MMR, a change identical to that observed for *U*_crit_. These findings suggest that the difference in *U*_crit_ was due, at least in part, to the improvement in respiratory capacity in the tributaries and the upper reach compared with the main stream or the lower stream.

Previous studies found that the swimming speed and 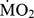 curve changed markedly as a consequence of the morphological divergence between fish living in different habitats. In this study, the 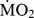-swimming speed curve still showed marked variation. The higher steepness of the 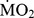-swimming speed curve of fish collected from the main stream and lower reach suggested a higher swimming cost based on an increase in speed compared with those from the tributary streams and the upper reach. The optimal *U*_crit_ values for the tributary streams and the upper reach were also higher than those for the main stream and lower reach, suggesting an adaptation to the water flow regime and (or) predator stress. However, the lower RMR of fish in lower reach or main stream might compensate for the higher swimming cost. This result suggests that an energy-saving strategy occurs in *O. biden*. In brook trout [30] and crucian carp [29]), individuals that showed higher energy expenditure during swimming also showed a lower RMR.

Most previous studies found a significant difference in unsteady swimming performance in fish living in different habitats [31], [32]. In many fish species, such as Trinidadian killifish (*Rivulus hartii*) [28] and Atlantic cod (*Gadus morhua*) [11], individuals that showed higher steady swimming performance tends to have lower unsteady swimming performance. However, no such trade-off was found at the population or individual levels in *O. bidens*. Note that such previous studies usually found marked morphological changes that favor either steady or unsteady swimming performance [33], [34]. However, even though one morphological parameter (fitness ratio) in *O. biden* was positively correlated with water velocity, neither morphological landmark points nor any calculated morphological parameters showed significant correlation with locomotor parameters. It suggested that in *O. biden*, the improved *U*_crit_ was a result of respiratory capacity rather than morphological divergence. Thus, the fish with the higher *U*_crit_ should not always show lower fast-start performance. The trade-off cost for higher steady swimming performance found for the fish in the upper reach and the tributary streams consists of a higher RMR and, hence, a greater routine energy expenditure rather than a lower fast-start capacity.

### Genetics

To our knowledge, this study is the first to measure morphology, genetics and swimming performance simultaneously in fish living along a river system. We aimed to test whether the morphological and locomotor divergences had a genetic base by comparing the genetic distances among different collecting sites. However, despite a substantial difference in the steady swimming speed among different reaches and between streams, only the fish from HK differed genetically from those found at the other collecting sites. This result suggests that the difference in the steady swimming speed may be due to phenotypic plasticity, at least for the collecting sites other than HK. However, this result is of interest because HK is only 50∼60 km from the main stream (YJ) and because this site included only one dam, which was built much later than most of the other dams in the study area. The mechanisms underlying this result need further investigation.

## Supporting Information

Figure S1 The average unwrap imagines and relative warps visualization plot of fish collected from different sites.
(PDF)

## References

[1] Langerhans RB (2009). Predictability of phenotypic differentiation across flow regimes in fishes.. Integrative and Comparative Biology.

[2] Plaut I (2001). Critical swimming speed: its ecological relevance.. Comparative Biochemistry and Physiology.

[3] Kieffer JD (2010). Perspective - Exercise in Fish: 50+ years and going strong.. Comparative Biochemistry and Physiology.

[4] Blake RW (1983). Fish locomotion. Cambridge: Cambridge University Press.. 208 p.

[5] Videler JJ (1993). Fish swimming. London: Chapman and Hall.. 260 p.

[6] Brett JR (1964). The respiratory metabolism and swimming performance of young sockeye salmon. Journal of the Fisheries Research Board Canada..

[7] Lee CG, Farrell AP, Lotto A, MacNctt MJ, Hinch SG (2003). The effect of temperature on swimming performance and oxygen consumption in adult sockeye (*Oncorhynchus nerka*) and coho (*O. kisutch*) salmon stocks.. The Journal of Experimental Biology.

[8] Webb PW, Blake RW, Hildebrand M, Bramble DM, Liem KF, Wake DB (1985). Swimming..

[9] Law TC, Blake RW (1996). Comparison of the fast-start performances of closely related, morphologically distinct threespine sticklebacks (*Gasterosteus spp.*).. The Journal of Experimental Biology.

[10] Walker JA (1997). Ecological morphology of lacustrine threespine stickleback *Gasterosteus aculeatus* L. (Gasterosteidae) body shape.. Biological Journal of the Linnean Society.

[11] Reidy SP, Kerr SR, Nelson JA (2000). Aerobic and anaerobic swimming performance of individual Atlantic cod.. The Journal of Experimental Biology.

[12] Blake RW (2004). Fish functional design and swimming performance.. Journal of Fish Biology.

[13] Langerhans RB, Elewa AMT (2006). Evolutionary consequences of predation: avoidance, escape, reproduction, and diversification..

[14] Langerhans RB, Gifford ME, Joseph EO (2007). Ecological speciation in Gambusia fishes.. Evolution.

[15] Langerhans RB, Layman CA, Langerhans AK, Dewitt TJ (2003). Habitat-associated morphological divergence in two Neotropical fish species.. Biological Journal of the Linnean Society.

[16] Collin H, Fumagalli L (2011). Evidence for morphological and adaptive genetic divergence between lake and stream habitats in European minnows (*Phoxinus phoxinus*, Cyprinidae) Molecular Ecology.

[17] Hendry AP, Hudson K, Walker J. Räsänen AK, Chapman LJ (2011). Genetic divergence in morphology–performance mapping between Misty Lake and inlet stickleback.. Journal of Evolutionary Biology.

[18] Yan GJ, He XK, Cao ZD, Fu SJ (2012). The trade-off between steady and unsteady swimming performance in six cyprinids at two temperatures.. Journal of Thermal Biology.

[19] He XK, Cao ZD, Fu SJ (2011). Fast-start swimming performances of juvenile *Cyprinus carpio* and the effects of electrical stimulation parameters.. Chinese Journal of Ecology.

[20] Fu SJ, Brauner CJ, Cao ZD, Richards JG, Peng JL (2011). The effect of acclimation to hypoxia and sustained exercise on subsequent hypoxia tolerance and swimming performance in goldfish (*Carassius auratus*).. The Journal of Experimental Biology.

[21] Fu SJ, Zeng LQ, Li XM, Pang X, Cao ZD (2009). The behavioral, digestive and metabolic characteristics of fishes with different foraging strategies.. The Journal of Experimental Biology.

[22] Pang X, Cao ZD, Fu SJ (2011). The effects of temperature on metabolic interaction between digestion and locomotion in juveniles of three cyprinid fish (*Carassius auratus*, *Cyprinus carpio* and *Spinibarbus sinensis*).. Comparative Biochemistry and Physiology.

[23] Xiao W, Zhang Y, Liu H (2001). Molecular systematics of Xenocyprinae (Teleostei: Cyprinidae): taxonomy, biogeography, and coevolution of a special group restricted in East Asia.. Molecular Phylogenetics and Evolution.

[24] Larkin MA, Blackshields G, Brown NP, Chenna R, McGettigan PA (2007). Clustal W and Clustal X version 2.0.. Bioinformatics.

[25] Tamura K, Peterson D, Peterson N, Stecher G, Nei M (2011). MEGA5: Molecular evolutionary genetics analysis using maximum likelihood, evolutionary distance, and maximum parsimony methods.. Molecular Biology and Evolution.

[26] Nelson JA, Gotwalt PS, Snodgrass JW (2003). Swimming performance of blacknose dace (*Rhinichthys atratulus*) mirrors home-stream current velocity.. Canadian Journal of Fisheries and Aquatic Science.

[27] McGuigan K, Franklin CE, Moritz C Blows MW (2003). Adaptation of rainbow fish to lake and stream habitats.. Evolution.

[28] Oufiero CE, Walsh MR, Reznick DN, Garland T (2011). Swimming performance trade-offs across a gradient in community composition in Trinidadian killifish (*Rivulus hartii*).. Ecology.

[29] Pettersson LB, Hedenström A (2000). Energetics, cost reduction and functional consequences of fish morphology.. Proceeding of Royal Society.

[30] Rouleau S, Glémet H, Magnan P (2010). Effects of morphology on swimming performance in wild and laboratory crosses of brook trout ecotypes.. Functional Ecology.

[31] Domenici P, Turesson H, Brodersen K, Brönmark C (2008). Predator-induced morphology enhances escape locomotion in crucian carp. Proceedings of Royal Society..

[32] Langerhans RB (2009). Morphology, performance, fitness: functional insight into a post-Pleistocene radiation of mosquitofish.. Biology Letter.

[33] Webster MM, Atton N, Hart PJB, Ward AJW (2011). Habitat-specific morphological variation among threespine sticklebacks (*Gasterosteus aculeatus*) within a drainage basin.. PLoS ONE.

[34] Varian A, Nichols KM (2010). Heritability of Morphology in Brook Trout with Variable Life Histories.. PLoS ONE.

