# Supplementary material for: Habitat-Specific Locomotor Variation among Chinese Hook Snout Carp (Opsariichthys bidens) along a River
Source: PLoS One. 2012 Jul 19;7(7):e40791. doi: 10.1371/journal.pone.0040791 (PMC3400668; doi:10.1371/journal.pone.0040791)
Supplement: Figure S1 — The average unwrap imagines and relative warps visualization plot of fish collected from different sites. (PDF) [file pone.0040791.s001.pdf]

Fig S1 The average unwrap imagines and relative warps visualization plot of fish collected from different sites.

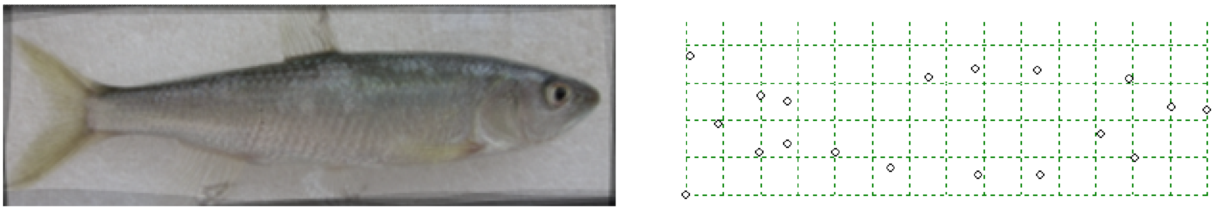

Daguan

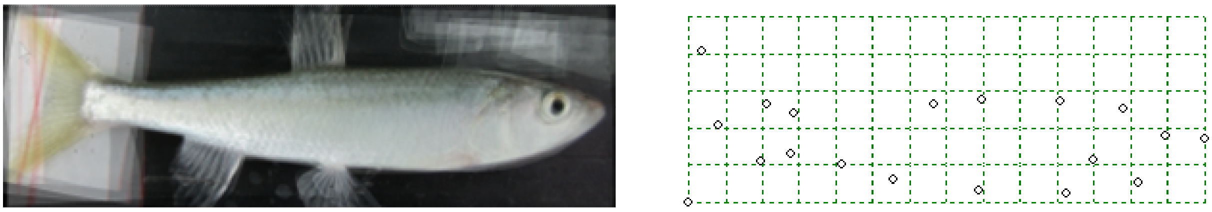

Haokou

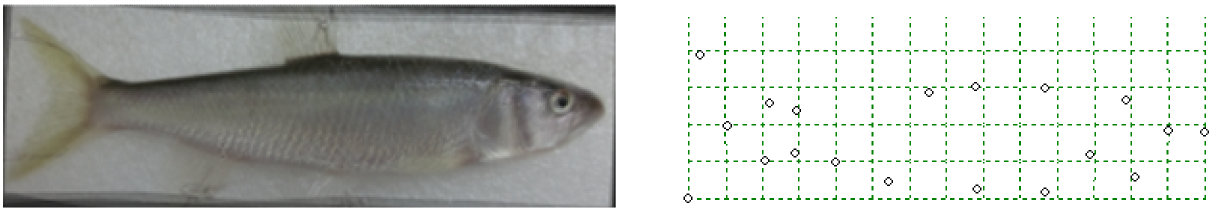

Sinan

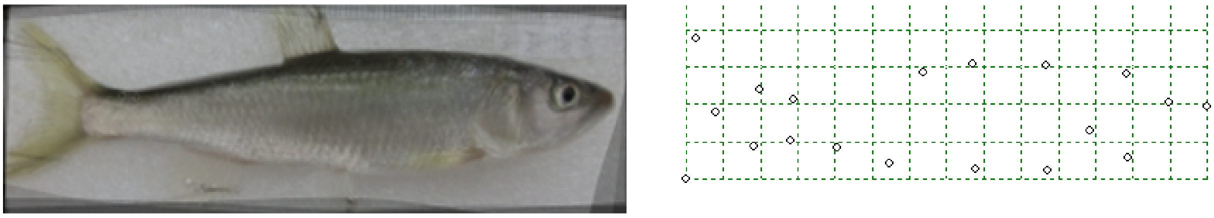

Sandu

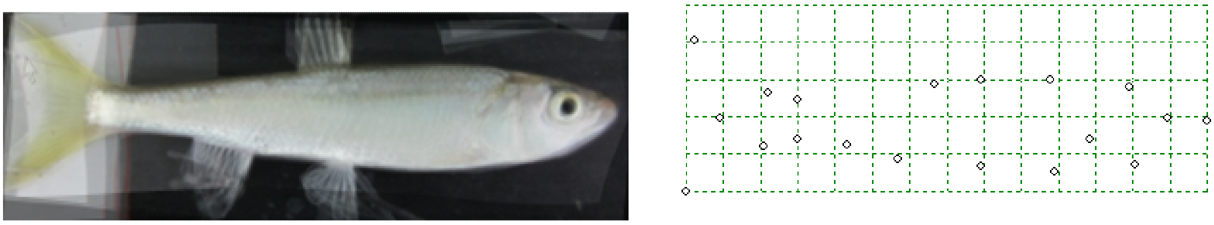

Yangjiao
